# Population pharmacokinetic and pharmacogenetic analysis of nevirapine in hypersensitive and tolerant HIV-infected patients from Malawi

**DOI:** 10.1186/1758-2652-13-S4-P181

**Published:** 2010-11-08

**Authors:** L Dickinson, M Chaponda, D Carr, JJ van Oosterhout, J Kumwenda, DG Lalloo, M Pirmohamed, R Heyderman, SH Khoo

**Affiliations:** 1 NIHR Biomedical Research Centre, Royal Liverpool & Broadgreen University Hospital Trust, Liverpool, UK; 2 University of Liverpool, Department of Pharmacology, Liverpool, UK; 3 University of Malawi, Department of Medicine, College of Medicine, Blantyre, Malawi; 4 Liverpool School of Tropical Medicine, Liverpool, UK

## Purpose of the study

Despite risk of hypersensitivity (HS), nevirapine (NVP) underpins first-line HIV therapy in Africa. The relationship between NVP exposure and HS is unknown but could be influenced by polymorphisms in CYP2B6 and CYP3A4 affecting drug metabolism.

## Methods

180 HIV patients (101 female) from Malawi receiving NVP-based therapy (200mg twice daily) between March 2007-September 2008 for a median (range) 6weeks (1-26) were included in the population pharmacokinetic (PK) model (n=383 NVP serum concentrations). Rich and sparse (n=40 and 140 patients, respectively) sampling was performed. Median (range) age, weight, BMI and CD4 cell count were 34yr (21-62), 54kg (35-94), 20kg/m^2^ (15-38) and 156cells/mm^3^ (1-812). In total 25 individuals were HS and 23 hepatitis B/C co-infected. Pharmacogenetic data were available for single nucleoside polymorphisms (SNPs) CYP3A5*6, CYP3A5*3, CYP2B6 983T>C, CYP2B6 516G>T, CYP2B6 785A>G in 89/180 patients obtained by Sequenom iPLEX. NONMEM (VI 2.0) was applied to determine NVP PK parameters, interindividual, interoccasion variability (IIV, IOV), residual error and influence of patient demographics, HS and genetics on NVP apparent oral clearance (CL/F). A visual predictive check was used to validate the model.

## Summary of results

A one compartment model with first order absorption best described NVP concentrations. For the final model (n=89) NVP CL/F (relative standard error; RSE%) was 2.67 (5%) with IIV and IOV of 30% (29%) and 32% (26%), respectively. Apparent volume of distribution and absorption rate constant were 141L (22%) and 0.77h^-1^ (31%), respectively. None of the patient demographics were significantly related to NVP CL/F. No association between NVP CL/F and HS or hepatitis infection was observed. Of the SNPs analysed CYP2B6 983T>C and CYP3A5*3 had a significant impact on NVP CL/F; reducing it by 25% in 983C heterozygotes (allelic frequency 18%) and 40% in CYP3A5*3 homozygotes (allelic frequency 5%).

## Conclusions

Available patient demographics and development of HS were not associated with NVP CL/F in this population of HIV-infected patients from Malawi. Genes associated with loss of function in CYP2B6 and CYP3A5 reduced NVP CL/F. However, NVP exposure was not associated with the development of HS, which is more likely to be an immunological phenomenon.

